# Designing multi-epitope-based vaccine targeting surface immunogenic protein of *Streptococcus agalactiae* using immunoinformatics to control mastitis in dairy cattle

**DOI:** 10.1186/s12917-022-03432-z

**Published:** 2022-09-07

**Authors:** Rajesh Kumar Pathak, Byeonghwi Lim, Do-Young Kim, Jun-Mo Kim

**Affiliations:** Department of Animal Science and Technology, Chung-Ang University, Gyeonggi-do 17546 Anseong-si, Republic of Korea

**Keywords:** Cattle, Mastitis, Multi-epitope vaccine, Epitope prediction, Immunoinformatics

## Abstract

**Background:**

Milk provides energy as well as the basic nutrients required by the body. In particular, milk is beneficial for bone growth and development in children. Based on scientific evidence, cattle milk is an excellent and highly nutritious dietary component that is abundant in vitamins, calcium, potassium, and protein, among other minerals. However, the commercial productivity of cattle milk is markedly affected by mastitis. Mastitis is an economically important disease that is characterized by inflammation of the mammary gland. This disease is frequently caused by microorganisms and is detected as abnormalities in the udder and milk. *Streptococcus agalactiae* is a prominent cause of mastitis. Antibiotics are rarely used to treat this infection, and other available treatments take a long time to exhibit a therapeutic effect. Vaccination is recommended to protect cattle from mastitis. Accordingly, the present study sought to design a multi-epitope vaccine using immunoinformatics.

**Results:**

The vaccine was designed to be antigenic, immunogenic, non-toxic, and non-allergic, and had a binding affinity with Toll-like receptor 2 (TLR2) and TLR4 based on structural modeling, docking, and molecular dynamics simulation studies. Besides, the designed vaccine was successfully expressed in *E. coli.* expression vector (pET28a) depicts its easy purification for production on a larger scale, which was determined through in silico cloning. Further, immune simulation analysis revealed the effectiveness of the vaccine with an increase in the population of B and T cells in response to vaccination.

**Conclusion:**

This multi-epitope vaccine is expected to be effective at generating an immune response, thereby paving the way for further experimental studies to combat mastitis.

## Background

Cattle are known as one of the most important animal sources of nutrients and have a significant role in the development of society. Based on scientific evidence, cattle milk has been recognized as a complete food. Its milk and dairy products are important sources of micronutrients and macronutrients [1]. The quality and productivity of cattle milk depend on cattle health [2], geographical area, and diet. Maintaining cattle health during the emergence of new pathogens due to climate change is a challenging task for farmers. Many diseases that emerged in past years have had a direct impact on cattle health and are responsible for a decrease in milk quality and productivity [3]. Mastitis is a complex and highly deleterious disease that is responsible for a significant loss to the dairy industry [4]. Mastitis can be classified as subclinical or clinical. There are no visible indicators of inflammation at the subclinical level [5]. However, inflammation in the mammary gland and milk abnormalities have been reported in clinical mastitis. Pain, swelling, heat, and redness of the udder are also signs of mild or moderate clinical mastitis [6, 7]. The occurrence of mastitis is concerning as it can cause zoonoses and food toxin infections, which affect human health [8].

Several factors are responsible for the induction of mastitis; however, *Staphylococcus* and *Streptococcus* species, such as *S. aureus, S. agalactiae, S. dysgalactiae*, and *S. uberis*, are common pathogens that cause clinical mastitis. Although *S. aureus* causes low-grade mastitis, co-infections can worsen the condition and even result in mortality [9–11]. The use of antibiotics to manage mastitis is limited, as their regular use results in residual antibiotics in milk and the development of antibiotic resistance in bacteria [12–14]. Although antibiotics are the standard treatments for mastitis, alternative herbal and homeopathic treatments can also be used, but these take a long time to alleviate the disease. Owing to the increase in population and demand for milk, extensive assessments are needed to effectively cure mastitis. Vaccines can be administered to prevent mastitis. However, regardless of the vaccine employed, treatment is not always successful or cost-efficient, especially in dairy herds where mastitis is prevalent [15]. Multi-epitopes vaccine, rather than a single-unit vaccine candidate, is a novel approach as it comprises cost-effective vaccines that offer remarkable specificity and durability in a variety of situations and provide long-term protection to cattle.

Due to advances in genomic science and bioinformatics, multi-epitopes vaccines can be designed quickly. In* Streptococcus*, surface immunogenic protein (Sip) with a mass of 53 kDa was discovered through immunological screening of a genomic library. The sip gene, which produces Sip, was found to be 98% identical at the nucleotide level (1305 bp) in the tested strains of *Streptococcus agalactiae*. Such a finding indicates that this 434-amino-acid protein is conserved and can be described as immunogenic. Sip was also demonstrated to provide an effective immune response and protection against *Streptococcus agalactiae* infection [16, 17]. Therefore, Sip can be used to design multi-epitopes vaccine candidates against mastitis.

A new discipline for designing multi-epitope vaccines has recently emerged owing to advances in immunoinformatics. This discipline has enabled us to gain a better understanding of the host immune response, significantly accelerating vaccine development. Herein, a multi-epitope vaccine against mastitis was designed using a variety of immunoinformatics tools to achieve effective protection against the disease. Sip epitopes (cytotoxic T-lymphocyte (CTL), helper T-lymphocyte (HTL), and B-cell epitopes) were predicted to be highly antigenic. Accordingly, to achieve maximum immune response, the current vaccine design includes all targeted epitopes conjugated with appropriate linkers and adjuvants. Various immunological and physicochemical properties of the multi-epitope vaccine were comprehensively assessed, and a 3D structural model of the vaccine was generated and analyzed.

**Fig. 1:**
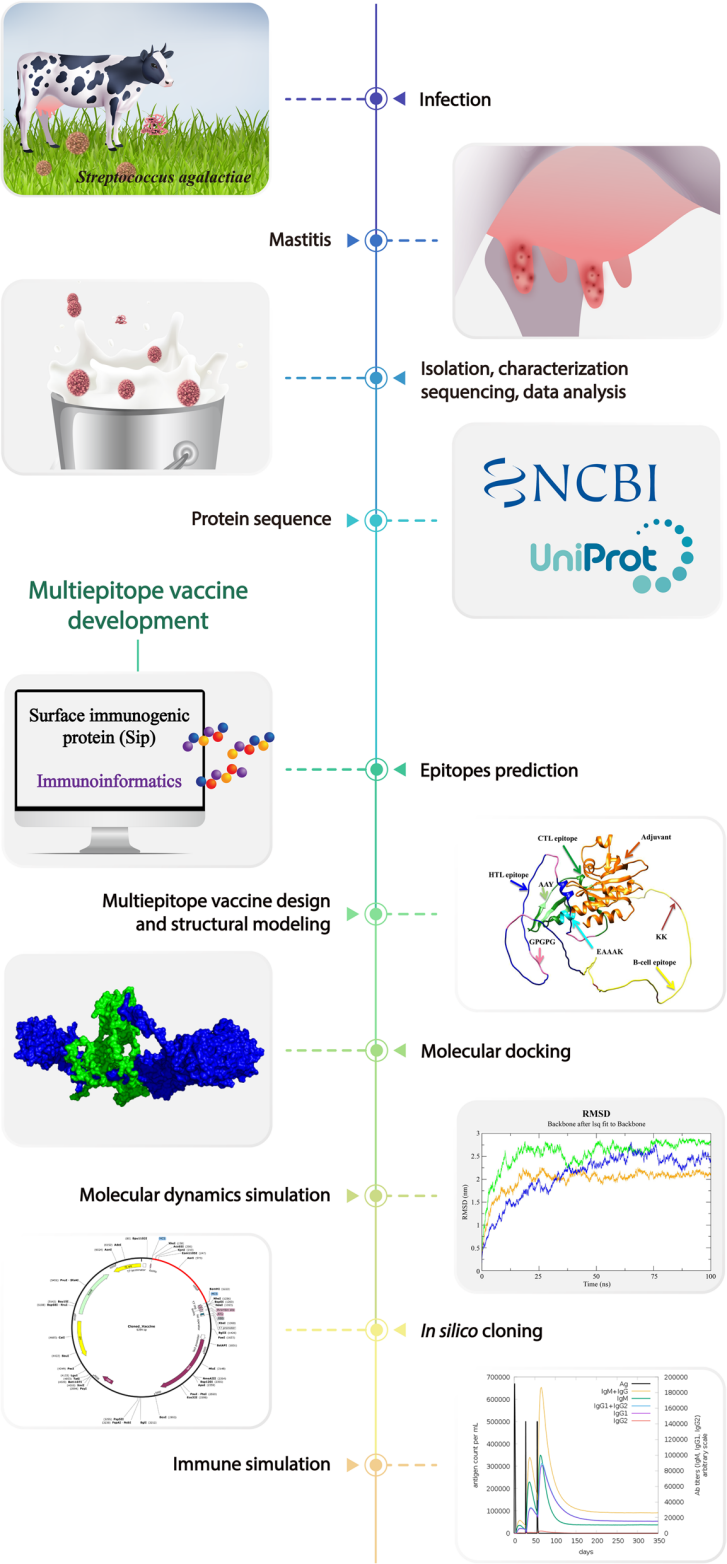
Schematic of mastitis infection in cattle, pathogen isolation, identification and availability of sequence information in public databases, and the immunoinformatics approaches for designing multi-epitope vaccine candidates

Molecular docking was used to assess the vaccine's binding affinity for Toll-like receptors (TLR) 2 and TLR4, and molecular dynamics (MD) simulation was used to reveal its stability and related interactions. Thereafter, the vaccine construct was cloned in silico in a prokaryotic expression system with codon optimization for large-scale manufacturing with improved translation efficiency followed by immune simulation analysis to determine the immune response and effectiveness of vaccine after vaccination (Fig. 1).

## Methods

### Sequence retrieval and antigenicity prediction

The amino acid sequence of Sip was retrieved from GenPept database (Accession no. CCW36894.1) of the National Center for Biotechnology Information (https://www.ncbi.nlm.nih.gov/protein) [18]. VaxiJen v2.0 was used to verify the antigenicity of Sip at a threshold of 0.4 (http://www.ddgpharmfac.net/vaxijen/VaxiJen/VaxiJen.html) [19]. Thereafter, epitope predictions were performed.

### Prediction of the CTL epitope

The presentation of antigen to CTL by Major Histocompatibility class I (MHC-I) is the first step in initiating an immune response against illnesses. The NetMHCpan 4.1 (https://services.healthtech.dtu.dk/service.php?NetMHCpan-4.1) server was used to predict CTL-epitopes using all available bovine leukocyte antigen (BoLA) alleles. The server relies on artificial neural networks to predict a peptide's affinity to bind to any MHC-I molecule in a known sequence. The affinities of epitopes were determined based on the highest prediction score and a % rank < 0.5 [20].

### Prediction of the HTL epitope

The HTL is recognized by MHC-II and plays an important role in the induction of cellular and humoral immune responses. Here, NetMHCIIpan 2.1 (https://services.healthtech.dtu.dk/service.php?NetMHCIIpan-2.1) server was used to predict the HTL epitopes. A traditional feed-forward artificial neural network was used to implement this server. This network is based on the NN-align method, which is a two-step procedure that calculates the peptide binding score (core) and network weight configuration [21]. The best epitopes with the highest binding affinity for the accessible BoLA class-II molecule were sorted using the lowest percentile rank score and a high prediction score.

### Prediction of B-cell epitope

The ABCpred (https://webs.iiitd.edu.in/raghava/abcpred/ABC_submission.html) server was used to predict B-cell epitopes. Briefly, the Sip sequence was used to determine linear B-cell epitopes that are unique, immunogenic, and continuous, with a threshold of 0.5. Epitope prediction is performed with high precision by the server using four parameters: sensitivity, specificity, positive predictive value, and accuracy [22].

### Design of the Multi-epitope vaccine

All selected epitopes of CTL, HTL, and B cells were joined using AAY, GPGPG, and KK linkers, respectively, to form a multi-epitope. An adjuvant Profilin (Uniprot ID- P02584) was added via the EAAAK linker to the N-terminus to improve immunogenicity. The final vaccine construct was 353 amino acids long after the addition of linkers and adjuvant [23].

### Similarity analysis

The final vaccine construct was subjected to NCBI BLASTp (https://blast.ncbi.nlm.nih.gov/Blast.cgi?PROGRAM=blastp&PAGE_TYPE=BlastSearch&LINK_LOC=blasthome) analysis against the non-redundant (nr) database of the bovine proteome to determine whether any similarities existed between them [24].

### Physicochemical and immunogenic properties prediction

The goal of vaccination is to provide an immunological response to the recipient. Therefore, vaccines should be stable, antigenic, non-allergic, and possess good solubility. VaxiJen v2.0 (http://www.ddg-pharmfac.net/vaxijen/VaxiJen/VaxiJen.html) was used to verify the antigenicity of the engineered multi-epitope vaccine [19]. AllerTOP v2.0 (https://www.ddg-pharmfac.net/AllerTOP/) and AllergenFP v1.0 (http://ddg-pharmfac.net/AllergenFP/) were used to screen for allergenicity [25]. Besides, physicochemical properties were evaluated using ProtParam (https://web.expasy.org/protparam/). Various parameters, such as molecular weight, theoretical pI, estimated half-life, instability index, aliphatic index, and grand average of hydropathicity (GRAVY), were evaluated in the study [26].

### Structure prediction, validation, visualization, and analysis

AlphaFold2 was used to predict the 3D model of an engineered multi-epitope vaccine. This powerful deep learning method is used to predict protein structure using sequence information with high accuracy [27, 28]. 3Drefine server (http://sysbio.rnet.missouri.edu/3Drefine/) was used to refine the predicted model [29]. The structural analysis and verification (SAVES) server (https://saves.mbi.ucla.edu/) was used to assess the quality of the predicted model. The predicted structure was visualized using UCSF Chimera [30]. Further, CABS-flex 2.0 (http://biocomp.chem.uw.edu.pl/CABSflex2) was used to determine the flexibility of the vaccine model [31].

### Molecular docking

To obtain an effective immune response, the vaccine must interact well with the host's immunological receptors. Therefore, protein–protein docking was used to predict the interaction of multi-epitope vaccine with immune receptors, TLR2 and TLR4. The 3D structure of TLR2 (AF-Q95LA9-F1) and TLR4 (AF-Q9GL65-F1) of bovine was retrieved from AlphaFold Protein Structure Database (https://alphafold.ebi.ac.uk/) [32]. ClusPro 2.0 (https://cluspro.bu.edu/login.php) server was employed for protein–protein docking. PyMOL (https://pymol.org/2/) and PDBsum (http://www.ebi.ac.uk/thornton-srv/databases/pdbsum/Generate.html) was used to analyze and visualize docked complex structures.

### Molecular dynamics (MD) simulation

The Gromacs 2018.1 (GROningen MAchine for Chemical Simulations) package was used to run MD simulations to further assess the stability of the vaccine and docked vaccine complexes [33]. The topology files were generated using the GROMOS96 53a6 force field [34]. To reduce steric hindrance, the systems were neutralized and subjected to the steepest energy minimization to generate a maximal force below 1000 kJ/mol/nm. Long-range electrostatic interactions were determined using the particle mesh Ewald (PME) method [35]. For Lennard–Jones and Coulomb interactions, a radius cut-off of 1.0 nm was used. Further, the LINCS method was used for H-bond length constraints [36]. The PME approach with 1.6-Fourier grid spacing was used to assess long-range electrostatics, whereas a 10-cut-off distance was used to predict short-range non-bonded interactions [35]. Shake algorithms were used to fix all bonds, including H-bonds. System neutralization was then performed via the addition of charged ions. Further, energy minimization was conducted and minimized structure was produced [37]. NVT and NPT equilibration was conducted to maintain the volume, temperature, and pressure of the system. Finally, a 100 ns MD simulation was carried out for trajectory analysis [33, 38].

### Codon optimization and in silico cloning

The initial sequence of the vaccine construct was submitted to the Java Codon Adaptation Tool (JCat) (http://www.jcat.de/Start.jsp) for reverse translation and codon optimization in the *E. coli* host strain K12 to optimize the expression rate of a designed vaccine in an appropriate expression vector [39]. The GC content and the codon adaptation index (CAI) were analyzed to evaluate the transcription and translation efficiencies [40]. Further, restriction sites for BamH1 and XhoI at the N- and C-terminals were added to enable restriction cloning into the pET‐28a ( +) vector using the SnapGene tool (https://www.snapgene.com/).

### Immune response simulation

The vaccine construct's sequence was submitted to the C-ImmSim (https://kraken.iac.rm.cnr.it/C-IMMSIM/) for analysis of the immune response. C-ImmSim determines the humoral and cellular response of a mammalian immune system concerning vaccines [41, 42]. The ideal interval between vaccine shots is generally four weeks recommended for most vaccines currently in use. Therefore, the entire simulation ran for 1050 simulation steps during the course of three consecutive injections with time steps of 1, 84, and 168 (Where 1-time steps = 8 h). The default settings for the other simulation parameters were used [42–44].

## Results

### Protein selection and antigenicity evaluation

In *Streptococcus* species, Sip, a 434 amino acid conserved protein, is described as an immunogenic protein and has received remarkable attention for the design of a new protein-based vaccine. The sequence of Sip was retrieved and subjected to VaxiJen v2.0 to evaluate its antigenic potential. The overall prediction for the protective antigen score for Sip was 0.6753, at a threshold of 0.4, indicating a high probability of antigenicity. Thus, when used to construct multi-epitope vaccine, it has proven to be antigenic.

### Prediction and analysis of the CTL and HTL epitopes

CTL epitopes of 9–12 mer length were first predicted owing to their high binding affinity with various BoLA class-I alleles. The corresponding epitopes were labeled as "strong interactions" based on a high prediction score and the lowest percentile rank achieved against the BoLA alleles. The antigenicity score of the epitopes was also predicted. The selected MHC-I CTL epitopes and their position in an amino acid sequence of Sip, antigenicity score, affinity with BoLA alleles, prediction score, and % rank are provided in Table 1.

**Table 1:** List of the selected cytotoxic T-lymphocyte (CTL) epitopes and their interacting BoLA class-I alleles with binding information

S.N	**MHC-I epitopes**	**Position**	**Antigenicity**	**Binding alleles**	**Prediction score**	**% Rank**
1	GLQPHVAAY	323–331	1.4739	BoLA-2:06,201	0.585	0.06
				BoLA-1:00,901	0.819	0.07
				BoLA-T5	0.533	0.08
				BoLA-1:00,902	0.533	0.08
				BoLA-2:06,901	0.186	0.13
				BoLA-1:03,101	0.404	0.15
				BoLA-D18.4	0.650	0.18
				BoLA-1:02,301	0.650	0.18
				BoLA-4:02,402	0.619	0.18
				BoLA-1:03,102	0.272	0.19
				BoLA-4:02,401	0.401	0.29
				BoLA-2:05,501	0.190	0.33
				BoLA-2:00,802	0.214	0.36
				BoLA-2:04,401	0.379	0.39
				BoLA-2:06,001	0.111	0.40
				BoLA-2:00,801	0.303	0.45
				BoLA-2:04,402	0.176	0.45
2	NKSSYTVKY	49–57	1.3394	BoLA-D18.4	0.688	0.15
				BoLA-T5	0.519	0.09
				BoLA-1:00,902	0.519	0.09
				BoLA-1:02,301	0.688	0.15
				BoLA-1:03,101	0.330	0.26
				BoLA-1:03,102	0.222	0.32
				BoLA-1:04,201	0.301	0.21
				BoLA-2:02,201	0.255	0.10
				BoLA-2:04,301	0.262	0.09
				BoLA-2:04,601	0.223	0.32
				BoLA-2:04,801	0.559	0.13
				BoLA-2:05,501	0.498	0.01
				BoLA-2:06,201	0.373	0.33
				BoLA-2:06,901	0.138	0.29
				BoLA-3:03,701	0.529	0.04
				BoLA-3:05,101	0.186	0.29
3	SVADQKVSL	132–140	1.0980	BoLA-HD6	0.378	0.77
				BoLA-JSP.1	0.271	0.76
				BoLA-T2c	0.981	0.01
				BoLA-T7	0.555	0.00
				BoLA-1:02,801	0.154	0.36
				BoLA-1:06,101	0.464	0.05
				BoLA-1:06,701	0.531	0.10
				BoLA-2:00,501	0.438	0.06
				BoLA-2:00,601	0.138	0.38
				BoLA-2:00,602	0.164	0.41
				BoLA-2:01,602	0.138	0.38
				BoLA-2:03,001	0.213	0.06
				BoLA-2:04,701	0.201	0.45
				BoLA-2:05,401	0.081	0.05
				BoLA-2:05,601	0.179	0.12
				BoLA-2:05,701	0.509	0.05
				BoLA-2:06,001	0.130	0.29
				BoLA-2:06,901	0.156	0.23
				BoLA-2:07,001	0.192	0.25
				BoLA-3:00,102	0.067	0.33
				BoLA-3:00,103	0.146	0.12
				BoLA-3:01,001	0.284	0.10
				BoLA-3:03,501	0.037	0.32
				BoLA-3:03,601	0.514	0.02
				BoLA-3:03,801	0.268	0.07
				BoLA-3:05,002	0.211	0.40
				BoLA-3:05,201	0.418	0.04
				BoLA-3:06,501	0.427	0.37
				BoLA-3:06,601	0.526	0.04
				BoLA-3:06,602	0.504	0.06
				BoLA-3:06,801	0.464	0.05
				BoLA-3:07,301	0.473	0.01
				BoLA-4:06,301	0.260	0.08
				BoLA-5:00,301	0.454	0.12
				BoLA-5:03,901	0.197	0.14
				BoLA-5:06,401	0.195	0.05
				BoLA-5:07,201	0.321	0.04
				BoLA-6:01,501	0.119	0.26
				BoLA-6:01,502	0.075	0.27
4	TGASPEHV	260–267	1.0130	BoLA-3:01,701	0.595	0.04
				BoLA-3:01,702	0.540	0.02
				BoLA-3:01,703	0.527	0.04
5	SQAAANEQV	181–189	1.0004	BoLA-1:02,101	0.033	0.39
				BoLA-1:02,801	0.231	0.18
				BoLA-1:06,101	0.546	0.03
				BoLA-1:07,401	0.128	0.39
				BoLA-2:05,401	0.029	0.46
				BoLA-5:00,301	0.335	0.44

Herein, 15 mer HTL epitopes were predicted. Based on strong binding affinity with the distinct subtypes of BoLA DRB3 alleles, highest prediction score, and lowest percentile rank score, 4 of these epitopes were selected for multi-epitope vaccine design. The antigenicity nature of each epitope was also evaluated. The sequence of selected HTL epitopes, their position, antigenicity score, affinity with BoLA alleles, prediction score, and % rank are shown in Table 2.

**Table 2:** List of the selected helper T-lymphocyte (HTL) epitopes and their interacting BoLA MHC-II alleles with binding information

SN	Peptide MHC II	Position	Antigenicity	Binding alleles	Prediction score	% Rank
1	SEAMSIDMNVLAKIN	65–79	0.9694	BoLA-DRB3*0401	0.700	0.70
				BoLA-DRB3*0501	0.671	0.50
				BoLA-DRB3*0503	0.634	0.90
				BoLA-DRB3*0801	0.669	0.60
				BoLA-DRB3*1501	0.657	0.30
				BoLA-DRB3*1703	0.482	0.40
				BoLA-DRB3*1801	0.601	0.80
				BoLA-DRB3*1802	0.611	0.30
				BoLA-DRB3*1902	0.671	0.90
				BoLA-DRB3*2301	0.641	0.40
				BoLA-DRB3*2901	0.510	0.60
				BoLA-DRB3*3001	0.683	0.50
				BoLA-DRB3*3002	0.748	0.50
				BoLA-DRB3*3201	0.408	0.30
				BoLA-DRB3*3202	0.524	0.40
				BoLA-DRB3*3203	0.547	0.40
				BoLA-DRB3*3601	0.649	0.80
2	PVRTVAAPRVASVKV	239–253	0.8232	BoLA-DRB3*0101	0.805	2.00
				BoLA-DRB3*1103	0.705	4.00
				BoLA-DRB3*2005	0.624	8.00
				BoLA-DRB3*3501	0.618	2.00
3	MKTYSSAPALKSKEV	159–173	0.6597	BoLA-DRB3*0101	0.761	4.00
				BoLA-DRB3*0301	0.815	0.30
				BoLA-DRB3*0502	0.617	0.40
				BoLA-DRB3*0901	0.742	0.60
				BoLA-DRB3*0902	0.653	0.50
				BoLA-DRB3*1602	0.694	0.15
				BoLA-DRB3*2401	0.464	0.50
				BoLA-DRB3*2702	0.635	0.15
				BoLA-DRB3*2703	0.635	0.15
				BoLA-DRB3*2704	0.588	0.40
				BoLA-DRB3*2707	0.609	2.00
				BoLA-DRB3*2710	0.635	0.15
				BoLA-DRB3*4501	0.606	0.40
				BoLA-DRB3*46,011	0.505	0.80
				BoLA-DRB3*6201	0.696	5.00
4	STQNMAANNISYVIW	379–393	0.5100	BoLA-DRB3*0501	0.596	2.00
				BoLA-DRB3*1501	0.602	0.70
				BoLA-DRB3*1903	0.611	4.00
				BoLA-DRB3*2301	0.557	1.50
				BoLA-DRB3*3001	0.677	0.50
				BoLA-DRB3*3002	0.670	1.50

### Prediction and analysis of B-cell epitopes

Sip B-cell epitopes were predicted using the ABCPred tool. According to the highest ranking among all anticipated epitopes based on significant binding affinity to B-cell receptors, four epitopes were selected for further evaluation. To construct the multi-epitope vaccine, these epitopes were selected as potent B-cell epitopes. A list of the selected B-cell epitopes and their position, binding, and antigenicity score is provided in Table 3.

**Table 3:** List of the selected Linear B lymphocyte (LBL) epitopes, their position in Sip, binding, and antigenicity score

SN	LBL epitope	Position	Score	Antigenicity
1	SMKIETPATNAAGQTT	106–121	0.97	1.2947
2	TYRAGDPGDHGKGLAV	346–361	0.97	0.7940
3	TSEVPAAKEEVKPTQT	198–213	0.84	0.8108
4	ANTWNAMPDRGGVTAN	408–423	0.83	0.9184

### Design of final multi-epitope vaccine candidate

**Fig. 2:**
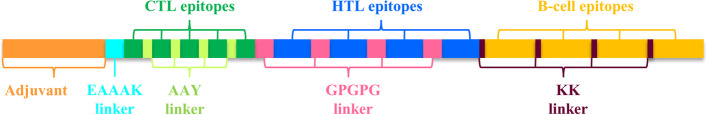
Illustration of the multi-epitope vaccine design. Adjuvants, linkers, and epitopes are shown in the final vaccine construct

For multi-epitope vaccine design, linkers were used to join all selected epitopes that could elicit a humoral and cell-mediated immune (CMI) response. AAY, GPGPG, and KK linkers were used to join five CTL epitopes, four HTL epitopes, and four B-cell epitopes. Further, EAAAK linker was used to adjoin profilin as an adjuvant at the N-terminal to generate a single construct with overall reactivity. Finally, a multi-epitope vaccine candidate with 353 amino acid residues was produced (Fig. 2).

### Assessment of the physicochemical and immunogenic properties of the vaccine

The non-homology of the constructed vaccine for the bovine host was first determined using NCBI BLASTp analysis. The vaccine design was demonstrated to be non-allergenic, non-toxic, and highly soluble, with an antigenic score of 0.7612 at a 0.4% threshold value using a VaxiJen tool. Thereafter, we proceeded to carry out a physicochemical analysis. The construct's molecular weight was 36 kDa, indicating its antigenicity and ease of purification. Proteins with a molecular mass of less than 110 kDa are easier to purify. Accordingly, these proteins are the best candidates for large-scale manufacturing. The peptide's basic nature is revealed by its pI value of 9.45. At 0.1% absorption, the extinction coefficient was 42,860, implying that all cysteine residues were reduced. The protein's half-life was > 30 h in human reticulocytes, > 20 h in yeast, and > 10 h in *E. coli*, implying that it can be exposed to the host for an extended period and stimulate the immune system. The construct's stability was also confirmed by an instability index of 34.73. The strong thermostability and hydrophilicity qualities were revealed by the aliphatic index of 68.36 and GRAVY (grand average of hydropathy) index of -0.295, indicating enhanced interactions within the body's polar environment. These findings suggest that the vaccine could be a good candidate for future vaccine development.

### Structural modeling, validation, and analysis of the vaccine model

**Fig. 3:**
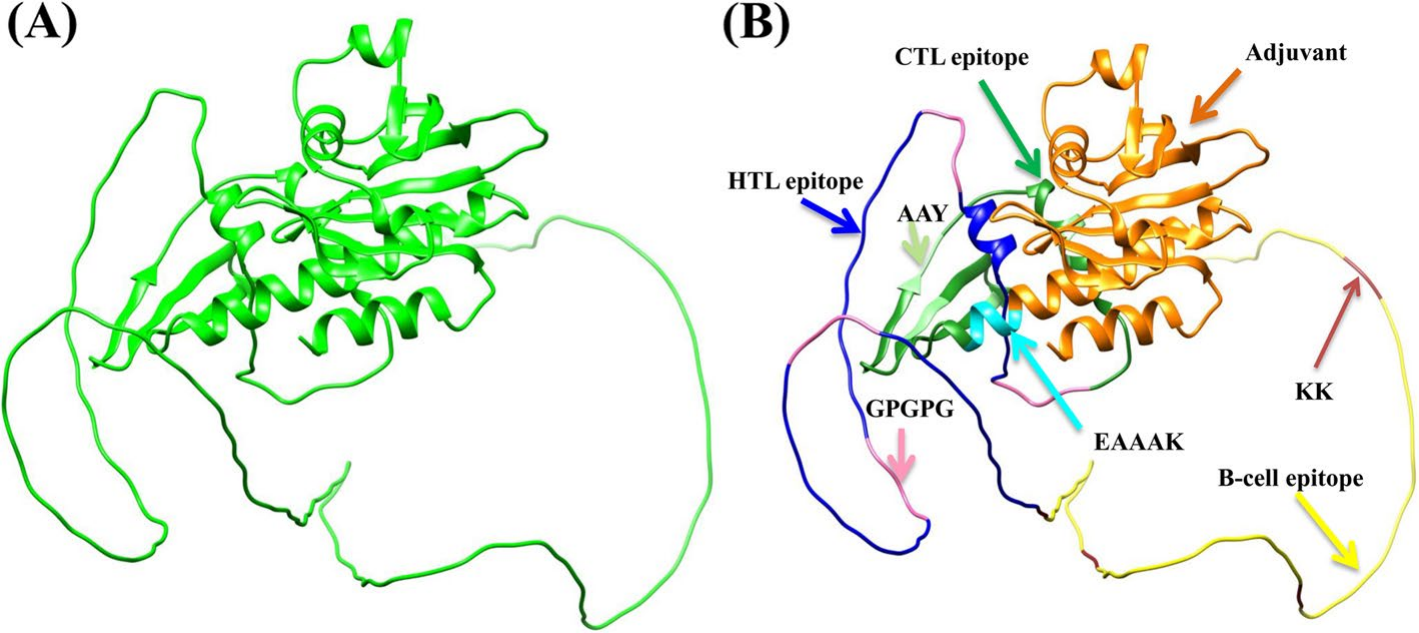
Structural modeling, **A** 3D structure of the refined multi-epitope vaccine model depicting helix, strand, and coil; **B** Adjuvant (Orange), EAAAK linker (Cyan), AAY linkers (Light green), GPGPG linkers (pink), KK linkers (Dark red) and epitopes, CTL (Forest green), HTL (Blue) and B-cell (Yellow) are shown in the multi-epitope vaccine 3D model

AlphaFold2 was used to model the 3D structure of the designed multi-epitope vaccine using deep learning method. Structural refinement was also conducted using the 3Drefine tool. The quality of the refined model was validated by the SAVES server (https://saves.mbi.ucla.edu/). The PROCHECK result based on Ramachandran plot analysis depicted 82.2% residues in the core or most favored region, 10.3% residues in the additional allowed region, 3.4% residues in the generously allowed region, and 4.1% residues in the disallowed region. These findings suggest that the quality of the refined model was good. Structure visualization was carried out by UCSF Chimera (Fig. 3).

**Fig. 4:**
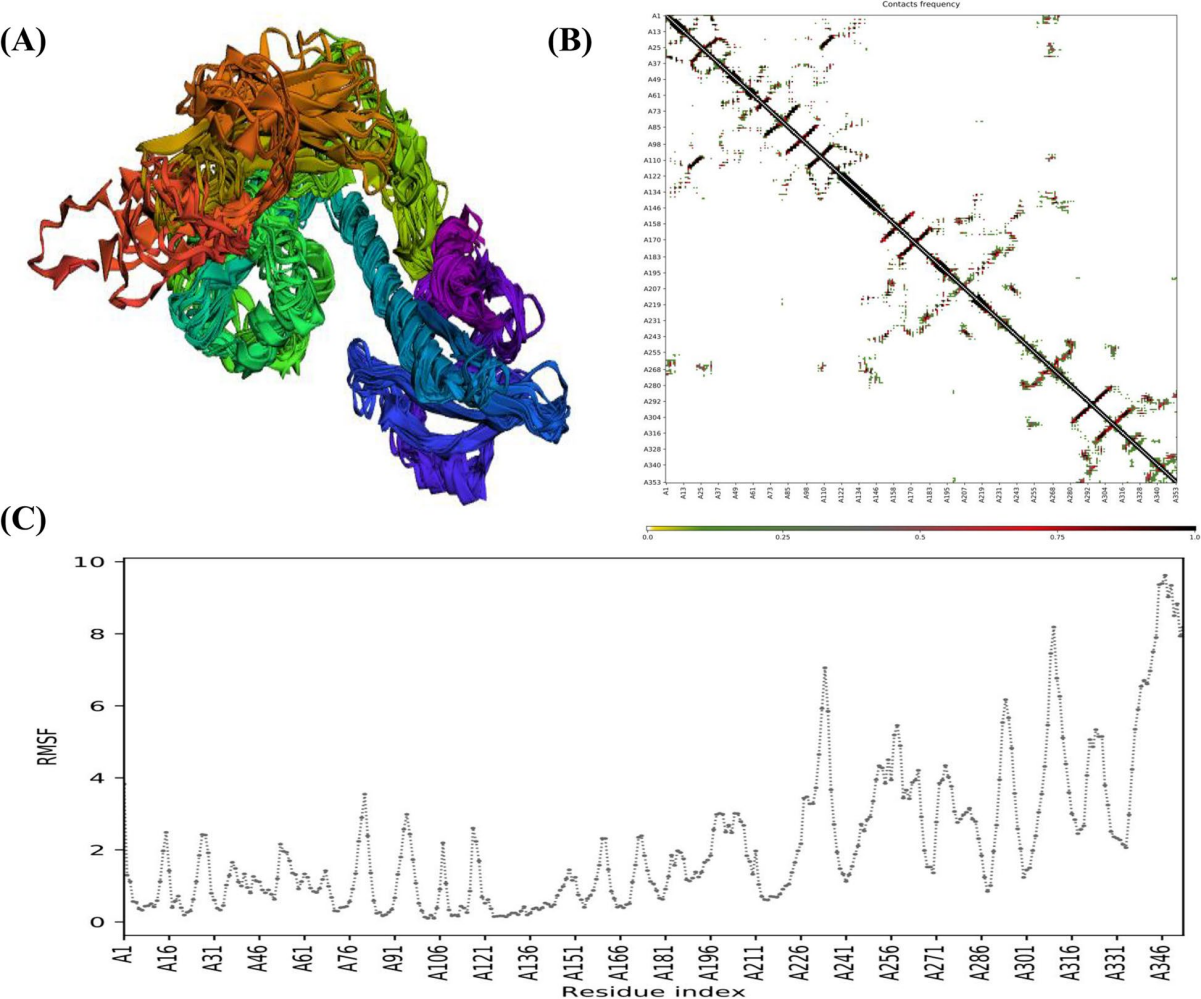
Analysis of structural flexibility. A Illustration of all ten models revealed fewer fluctuations, **B** Contacts map depicting residue-residue interaction, **C** RMSF plot depicting obvious fluctuations in amino acid residues determined during simulation

The vaccine's flexibility was assessed using the online program, CABS-flex 2.0, which ran 50 simulations at a default temperature of 1.4 °C. When the regions near the N-terminus are compared to those near the C-terminus, the collective model of ten retrieved structures revealed fewer variations. Contacts between distinct residues of all ten final retrieved structures are depicted in the contact map. Finally, the fluctuation plot reflected each amino acid's Root Mean Square Fluctuations (RMSF) (Fig. 4). Such differences in the designed vaccine construct's RMSF demonstrate its high flexibility, supporting its potential for application as a possible vaccine.

### Molecular docking of vaccine with TLR2 and TLR4

**Fig. 5:**
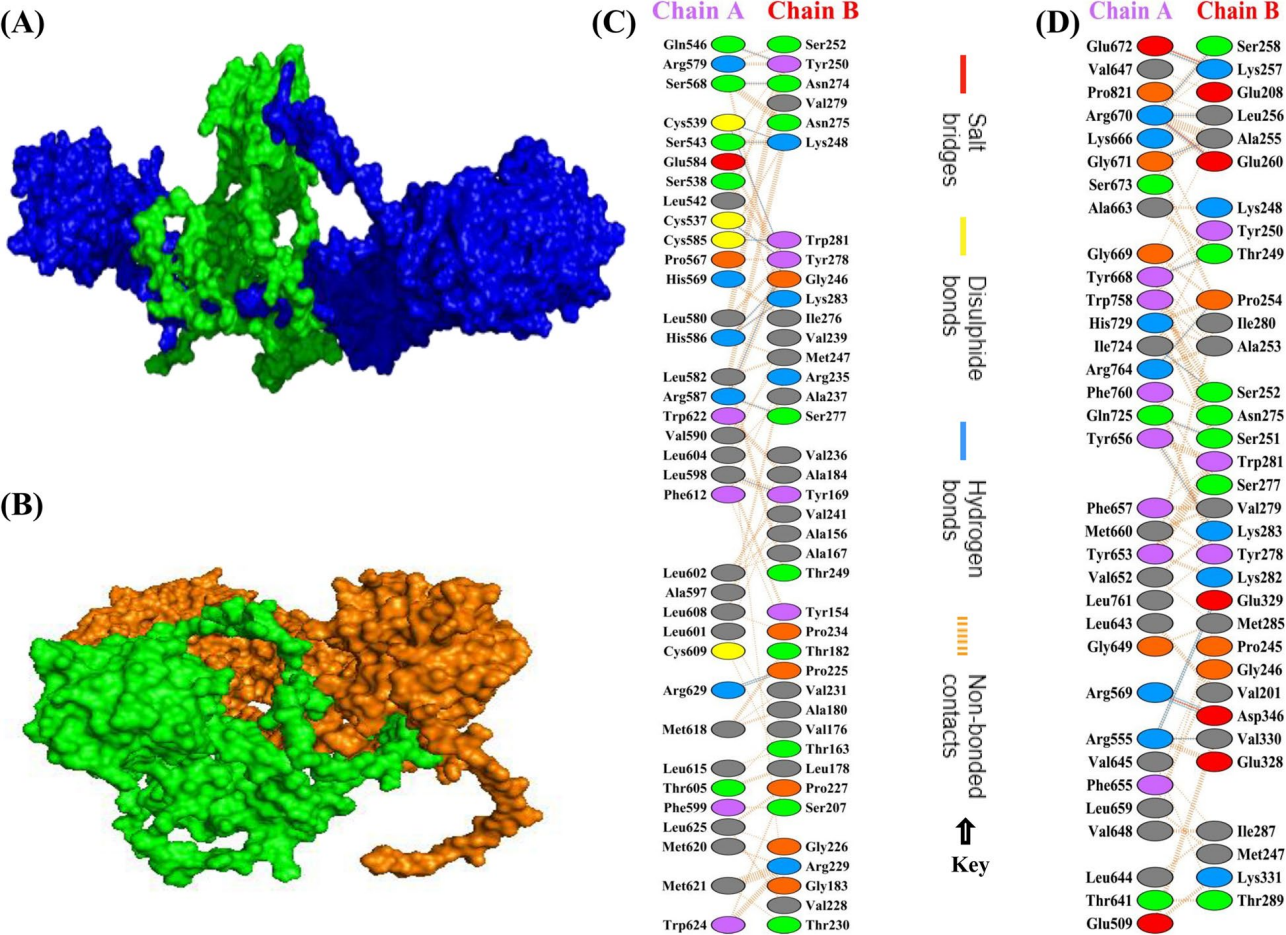
Molecular docking studies. **A** Docked complex of multi-epitope vaccine (Green) and TLR2 (Blue), **B** Docked complex of multi-epitope vaccine (Green) and TLR4 (Orange), **C** Interacting amino acid residues between TLR2 (Chain A) and multi-epitope vaccine (Chain B), **D** Interacting amino acid residues between TLR4 (Chain A) and multi-epitope vaccine (Chain B)

ClusPro 2.0 was used to perform molecular docking of the designed multi-epitope vaccine against bovine TLR2 and TLR4. ClusPro 2.0 generated 30 docked structures. Of these, the model with the highest binding affinity and the lowest intermolecular energy was selected. During docking with TLR2 and TLR4, the lowest energy scores of -1581.3 and -1500.8 were predicted, respectively. Further, the docked vaccine-TLRs complex structure was analyzed and visualized by PyMOL and PDBsum (Fig. 5). Finally, the structure was subjected to MD simulation using Gromacs.

### MD simulation of vaccine and docked vaccine-TLRs complexes

**Fig. 6:**
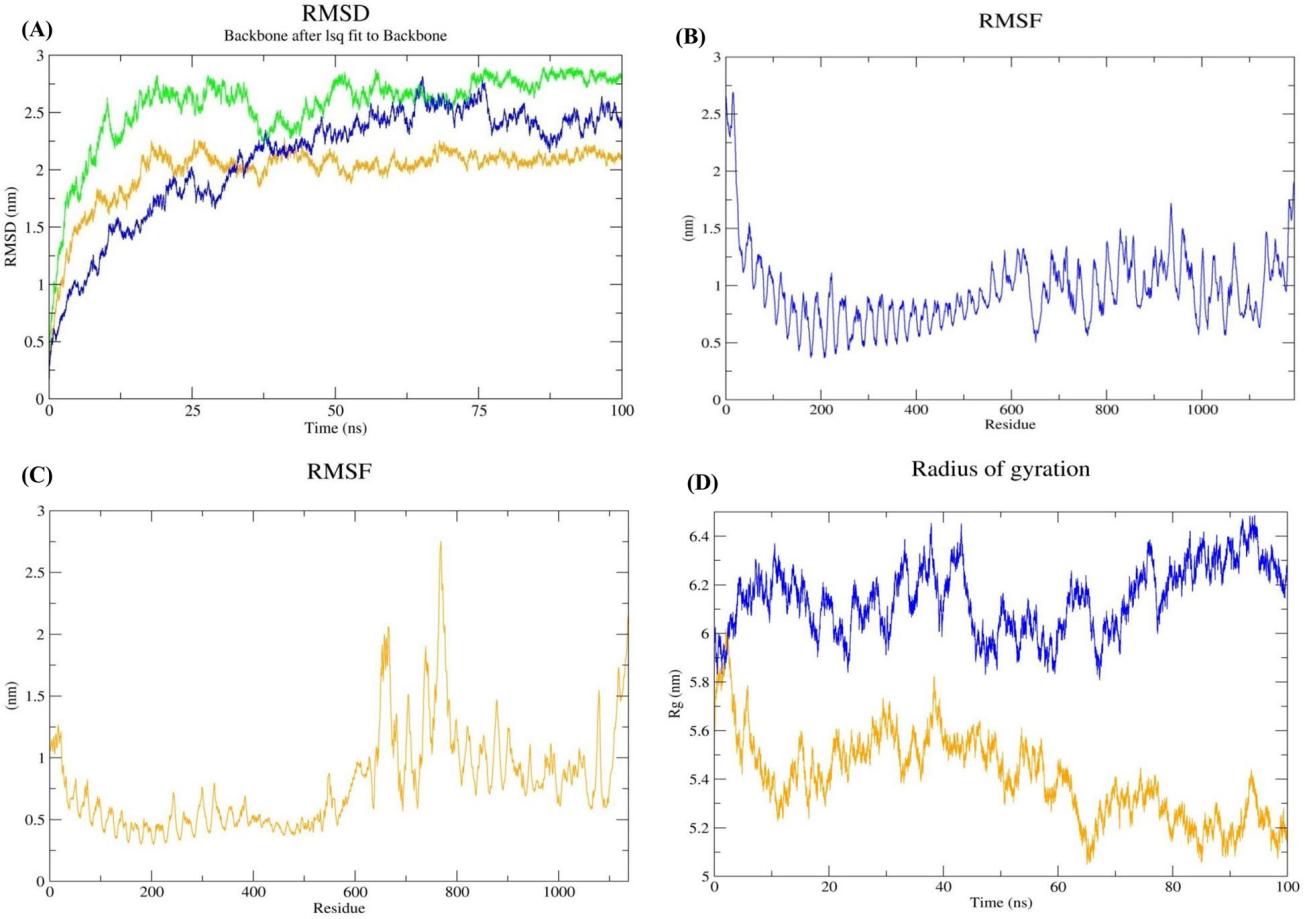
Molecular dynamics simulation analysis of vaccine-TLRs complexes. **A** RMSD plot of multi-epitope vaccine and vaccine-TLRs docked complexes **B** RMSF plot of vaccine-TLR2, **C** RMSF plot of vaccine-TLR4 and **D** Rg plot of vaccine-TLR2 and vaccine-TLR4 complex observed during 100 ns MD simulation. Green, blue, and orange represent vaccine, vaccine-TLR2 complex, and vaccine-TLR4 complex, respectively

MD simulation is a prominent tool for analyzing protein structural reliability in a simulated environment that is similar to real-world systems. MD simulations were run for 100 ns using Gromacs to further validate the vaccine construct and docked vaccine-TLRs structural integrity. The structural stability of the designed vaccine model and docked vaccine-TLRs complexes was evaluated using root mean square deviation (RMSD) analysis. RMSD analysis is one of the important methods for describing the dynamic behavior among native structures to a new pose with respect to time by utilizing MD simulation data. Based on the entire 100 ns MD trajectory, the RMSD values of each frame were calculated and plotted against time. The results revealed that the initial deviation had an increasing trend in all three systems (i.e., multi-epitope vaccine model, vaccine-TLR2, and vaccine-TLR4) until 40–50 ns. After 75 ns of simulation, less fluctuation was observed, indicating the stability of all systems (Fig. 6A). Further, structural flexibility and compactness of docked vaccine-TLRs were analyzed through root mean square fluctuation (RMSF), and radius of gyration (Rg). The RMSF value ranged from 1 to 1.5 nm, and the higher values correspond to highly flexible regions in the vaccine-TLR2 complex (Fig. 6B). Besides, RMSF value ranged from 1.5 to 2.5 nm, and the higher indicates highly flexible regions in the vaccine-TLR4 complex (Fig. 6C), revealing the flexibility and stability of the complex. In addition, the vaccine–TRL complexes had lower fluctuations in Rg peak after 80 ns simulation time, indicating their compactness and stability (6D).

### In silico cloning of vaccine candidate

**Fig. 7:**
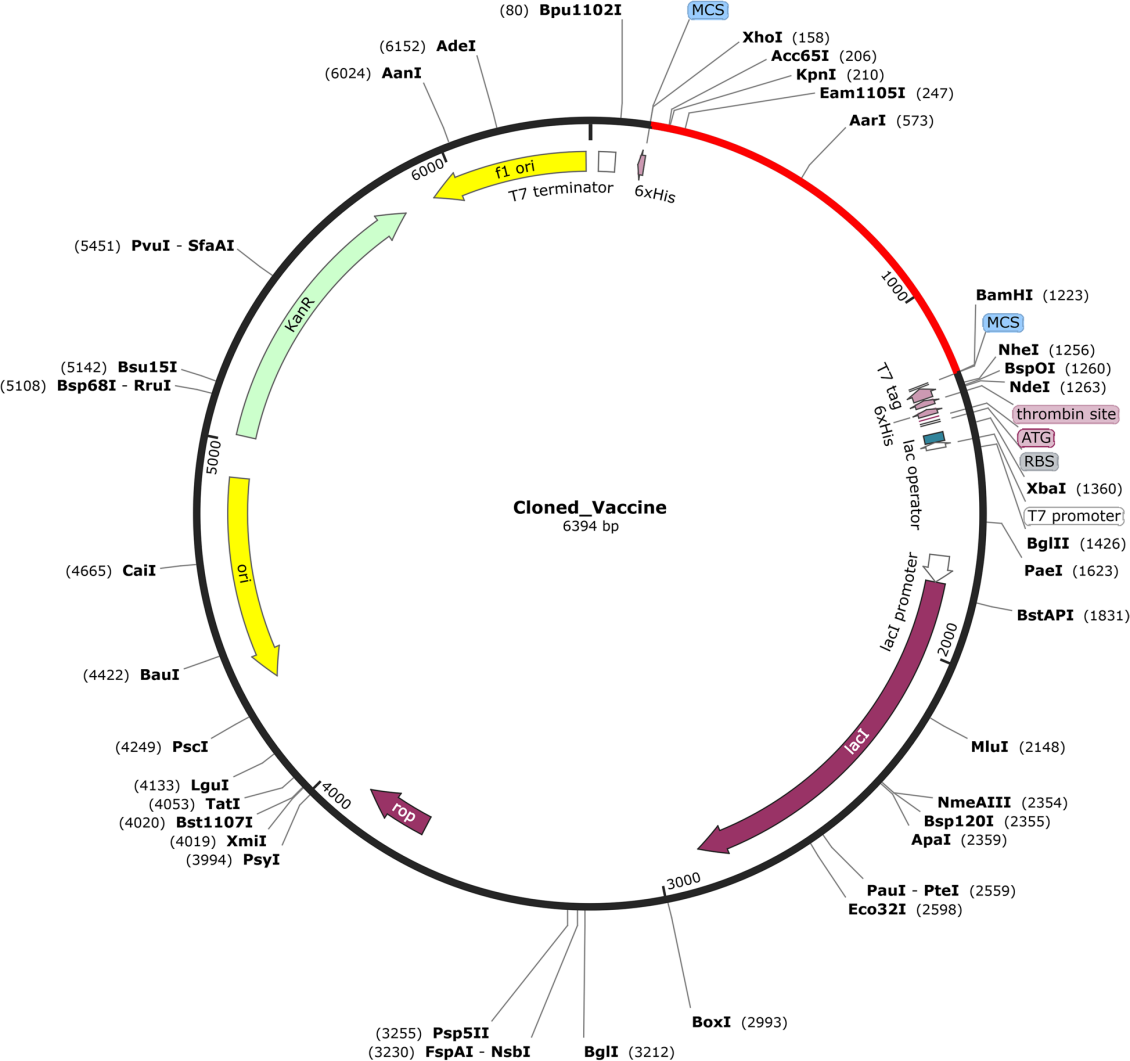
Multi-epitope vaccine constructs cloned into the pET28a( +) vector after codon optimization

*E. coli* K12 strains have a unique host expression system that requires codon adaptation. The codon adaptation index (CAI) of the optimized sequence is commonly used to represent this codon usage. The resultant cDNA had a CAI value of 1 and a GC content of 50.99%, indicating a high likelihood of expression in bacterial strain K12 based on the JCAT tool. For a high expression level, CAI of > 0.8 and GC content of 30–70% are desired. To ensure complementation in the vector translation direction, the optimized sequence was reversed, and BamHI and XhoI restriction sites were inserted at the 5’ and 3’ ends, respectively. SnapGene software was then used to insert this sequence into the pET28a ( +) expression vector. Finally, a recombinant plasmid was constructed with a sequence length of 6,394 bp that can be expressed successfully in the *E. coli* system (Fig. 7).

### Immune response simulation analysis

**Fig. 8:**
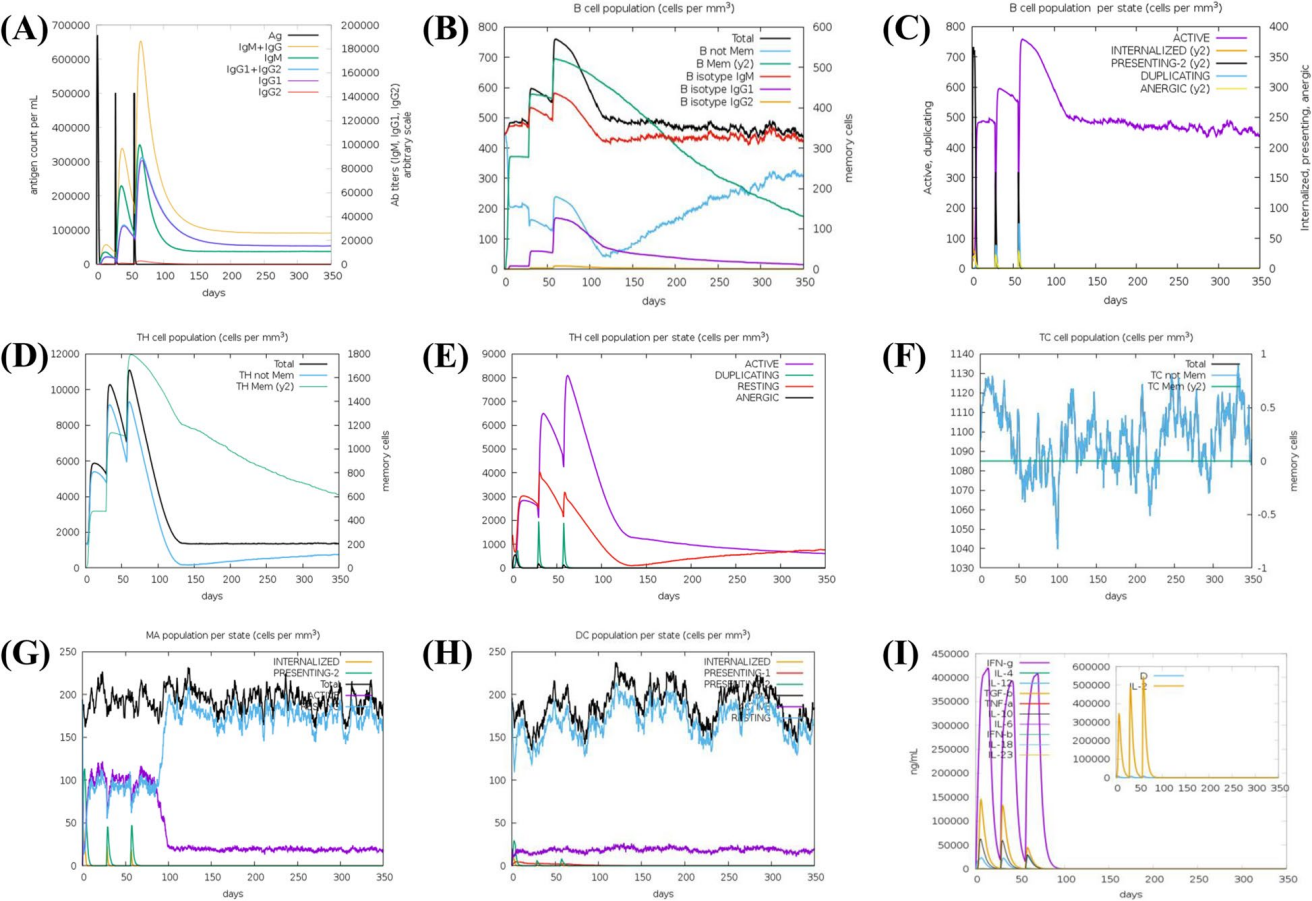
Vaccination-induced immune response triggered by designed multiepitope vaccine during immune simulation analysis **A** Levels of antibodies after primary, secondary and tertiary immune response, **B** B-cell population, **C** B-cell population per state, **D** helper T-cell population, **E** helper T-cell population per state, **F** cytotoxic T-cell population per state, **G** macrophage population per state, **H** dendritic cell population per state and **I** Cytokine and interleukins production

The multi-epitope vaccine's immunogenic profile is depicted in Fig. 8. The results of Immune simulation revealed that compared to the primary response, the secondary and tertiary responses were generated at significantly higher rates. The antibodies (IgM + IgG, IgM, IgG1 + IgG2, IgG1 and IgG2) levels were found to have significantly increased (Fig. 8A). Additionally, the designed vaccine demonstrated its effectiveness during simulation by accumulating increased B-cell (Fig. 8 B-C) and T-cell populations (Fig. 8 D-F). Besides, a rise in Th1 concentration was observed after each dose. Moreover, an increase in the number of macrophages (Fig. 8G) and dendritic cells (Fig. 8H) implies that antigen-presenting cells are efficient at processing and delivering antigens to CD4 + and CD8 + cells. Further, higher levels of several cytokines were also observed (Fig. 8I), indicating favorable immunological responses of the designed vaccine.

## Discussion

Milk consumption meets the fundamental needs of the body and is particularly good for bone formation and development [45]. Cattle milk is highly nutritious and is abundant in calcium, potassium, vitamins, and protein, among other minerals [46, 47]. The full potential of dairy cattle must be realized to meet the demands of an ever-increasing population. Several barriers, such as illnesses that affect milk supply, prevent the full utilization of milk [4, 48, 49]. Mastitis, a worldwide endemic illness affecting dairy cattle, is one of the leading causes of decreased milk production efficiency [50]. Mastitis is a major concern in the dairy industrial sector as it is linked to unhappy and stressed cattle, which ultimately lead to significant financial losses [4]. Antibiotics are rarely used to treat mastitis as residual levels in milk are harmful to humans. Further, continuous use of antibiotics leads to the development of antibiotic resistance in bacteria. Herbal and homeopathic remedies are useful against the disease, but take a longer time to exhibit a therapeutic effect. Therefore, vaccination is one of the best options to protect cattle from Mastitis. Vaccines are essential for stimulating immune responses and protecting against illnesses. Traditional vaccine development is time-consuming and expensive. Immunoinformatics-guided approaches provide us with a variety of computational resources, including tools and databases, that can be used to cost-effectively, precisely, and timely design an effective candidate vaccine [51].

A multi-epitope vaccine construct incorporating CTL, HTL, and B-cell epitopes connected to an adjuvant and linkers was constructed in this work. This vaccine construct was found to be efficient and could stimulate the host's innate and adaptive immune responses, making it a strong candidate for vaccine development. SARS-CoV-2, HIV, Ebola virus, Zika virus, Nipah virus, and other multi-epitope vaccines have been previously developed using immunoinformatics guided study. Owing to the encouraging results of these studies, researchers in veterinary science have opted to use this method to tackle diseases in livestock [52–56]. Therefore, to design an efficient multi-epitope vaccine, Sip was selected. Sip is an immunogenic protein that provides an effective immune response and protection against *Streptococcus* species. Its antigenic potential was analyzed and CTL, HTL, and B-cell epitopes were subsequently predicted [57].

In general, epitopes with high binding affinity for experimentally confirmed alleles are a suitable choice for incorporation in the multi-epitope vaccine design. A detailed investigation of all BoLA alleles of class I/II molecules was carried out for CTL and HTL epitope prediction. Few epitopes were identified as effective antigenic peptides with high affinity among the various BoLA-I molecules [57]. As a result, the highest-ranked epitopes (CTL and HTL) were selected for vaccine design processes, as determined by a highly conservative threshold recognized by BoLA class- I/II. Furthermore, the antigenicity of all selected CTL and HTL epitopes was determined, and linear B-cell epitopes with the highest score were selected. To design the multi-epitope vaccine, adjuvant and linkers were used with prioritized epitopes. The adjuvant was added to the N-terminus of the multi-epitope vaccine design, and epitopes were linked using the EAAAK, AAY, GPGPG, and KK linkers. Linkers are an important component of vaccines as they improve independent domain expression, folding, and stability [58, 59]. Adjuvants are used in vaccine design to improve efficacy, stability, and long-term viability [23, 60]. The primary goal of vaccination is to elicit a fast immune response with no or minimal side effects on the host's body. As a result, the complete amino acid sequence of the designed multi-epitope vaccine construct was evaluated against the bovine proteome using BLASTp, which revealed no similarities, establishing safety inside the host. Further analysis revealed that the vaccine is non-allergic, highly antigenic, and non-toxic, with high solubility and optimal physicochemical properties [57]. The 3D model of the designed multi-epitope vaccine was built by AlphaFold2, a deep learning-based tool, and a comprehensive evaluation was performed through structural refinement. The Ramachandran plot analysis revealed that the model had good quality [27]. In previous studies, several TLRs on the surface of immune cells were demonstrated to activate the innate immune response. As a result, molecular docking was used to assess the vaccine's interaction with TLR2 and TLR4 [23, 61]. The docking score revealed a strong binding affinity and a stable association between the docked protein–protein complexes, which was supported by MD simulations [53]. Therefore, the designed vaccine would activate TLRs, resulting in higher immunological responses in the host.

The inconsistency of mRNA codons is one of the challenges in vaccine design, and gene expression will vary between hosts. Thus, codon optimization is crucial for achieving better expression [57, 62]. The codon-optimized vaccine CAI value and GC content revealed a greater expression in the *E. coli* K12 strain. *E. coli* is the most desired and recommended system for bulk production of recombinant proteins, as revealed via previous research. In silico restriction cloning was carried out using the pET28( +) vector for easy purification and the manufacture of prospective candidate vaccines on a larger scale [57]. However, based on the immune simulation study, we can conclude that vaccines are effective at eliciting the immune response [42–44]. One of the criteria for being a successful vaccine candidate is the induction of B-cells and T-cells [63–65]. During immune simulation, we observed that the level of B-cells and T-cells increases with every injection and maintains their population level. Furthermore, a higher level of macrophages, dendritic cells, and cytokines makes vaccine constructs capable of establishing an antibacterial environment [66, 67]. The simulation analysis of the immune response produced by our designed vaccine confirmed that it would induce a proper immune response after exposure. Therefore, holistically, our integrated immunoinformatics approach would support the development of a vaccine against mastitis.

## Conclusion

To eliminate *S. agalactiae* infection, we must adopt new control measures, including the design and development of vaccine candidates. In this study, numerous immunoinformatics methods were employed to design a multi-epitope vaccine against mastitis using different T-cell (CTL and HTL) and B-cell epitopes and the extremely significant protein, Sip, which is defined as immunogenic. The designed multi-epitope vaccine elicited a high affinity and stable binding conformation, according to molecular docking and simulation analysis. This vaccine was predicted to be a good vaccine candidate based on its physicochemical and immunogenic properties, as well as immune response analysis. Overall, the designed multi-epitope vaccine could be effective at eradicating *S. agalactiae* infection.

## Data Availability

The protein sequence generated and/or analyzed during the current study are available in the NCBI- GenPept repository, Accession number: CCW36894.1.

## Abbreviations

Sip: Surface immunogenic protein
CTL: Cytotoxic T-lymphocyte
HTL: Helper T-lymphocyte
MHC: Major histocompatibility class
GRAVY: Grand average of hydropathicity
SAVES: The structural analysis and verification server
TLRs: Toll-like receptors
MD: Molecular dynamics
RMSD: Root-mean-square deviation
CAI: Codon adaptation index
JCat: Java codon adaptation tool
